# Recovery of metagenome-assembled genomes from the phyllosphere of 110 rice genotypes

**DOI:** 10.1038/s41597-022-01320-7

**Published:** 2022-06-01

**Authors:** Pin Su, Wisnu Adi Wicaksono, Chenggang Li, Kristina Michl, Gabriele Berg, Dan Wang, Youlun Xiao, Renyan Huang, Houxiang Kang, Deyong Zhang, Tomislav Cernava, Yong Liu

**Affiliations:** 1 State Key Laboratory of Hybrid Rice and Institute of Plant Protection, Hunan Academy of Agricultural Sciences, Changsha, 410125 China; 2 Graz University of Technology, Institute of Environmental Biotechnology, Graz, 8010 Austria; 3 Hunan Agricultural University, Changsha, 410128 China; 4 State Key Laboratory for Biology of Plant Diseases and Insect Pests, Institute of Plant Protection, Chinese Academy of Agricultural Sciences, Beijing, 100193 China; 5 China National Hybrid Rice R&D Center, Hunan Hybrid Rice Research Center, Changsha, 410125 China

**Keywords:** Metagenomics, Microbiome

## Abstract

The plant microbiota plays crucial roles in sustaining plant health and productivity. Advancing plant microbiome research and designing sustainable practices for agriculture requires in-depth assessments of microorganisms associated with different host plants; however, there is little information on functional aspects of many microorganisms of interest. Therefore, we enriched microorganisms from the phyllosphere of 110 rice genotypes and subjected them to shotgun metagenomic sequencing to reconstruct bacterial genomes from the obtained datasets. The approach yielded a total of 1.34 terabases of shotgun-sequenced metagenomic data. By separately recovering bacterial genomes from each of the 110 rice genotypes, we recovered 569 non-redundant metagenome-assembled genomes (MAGs) with a completeness higher than 50% and contaminations less than 10%. The MAGs were primarily assigned to *Alphaproteobacteria*, *Gammaproteobacteria*, and *Bacteroidia*. The presented data provides an extended basis for microbiome analyses of plant-associated microorganisms. It is complemented by detailed metadata to facilitate implementations in ecological studies, biotechnological mining approaches, and comparative assessments with genomes or MAGs from other studies.

## Abstract (summary)

**Table:** 

Measurement(s)	Metagenome
Technology Type(s)	Illumina Sequencing
Factor Type(s)	Genotype
Sample Characteristic - Organism	Oryza sativa
Sample Characteristic - Environment	agricultural field
Sample Characteristic - Location	China

## Background & Summary

Rice is one of the world’s most important staple foods, accounting for more than 20% of total caloric intake worldwide^1^. Adverse climatic conditions and a wide range of pathogens threaten food security by causing significant yield losses in rice production^2^. Agrochemicals currently provide the most reliable solution to secure rice production, but they also cause serious environmental damage in all major growing areas. Recent research focused on the plant microbiota has highlighted the potential of various microorganisms to increase sustainability of rice producing by replacing chemical pesticides and fertilizers^3,4^. It has been shown that certain bacteria that can be enriched in field-grown rice seeds protect their hosts from widespread diseases and that they are naturally transmitted across plant generations^3^. Complementary studies focusing on the role of the microbiota in nitrogen-use efficiency have demonstrated that certain rice genotypes are able to recruit beneficial microorganisms that substantially improve plant growth^4^. These findings were enabled by linking specific plant phenotypes to microbial functions that have long been neglected.

Deciphering further plant-microbiome interactions useful for agriculture will require adequate datasets to facilitate the identification of beneficial components within the plant microbiota^5^. Currently, most studies on the plant microbiome are based on amplicon sequencing of various microbial marker genes^6^, but the achievable resolution in terms of taxonomic and functional profiles of such analyses is limited compared to shotgun sequencing-based metagenomics^7^. Host-microbiome interactions often rely on specific functions that can be provided by adapted microorganisms^8^. Especially the recovery metagenome-assembled genomes (MAGs), provides a valuable basis for genome-centric, functional analyses^9^. Advances in sequencing technologies and bioinformatic data processing have facilitated the development of methods that allow holistic analyses of microorganisms that have not yet been cultivated. Our aim was to further expand the resource base for phyllosphere microbiome studies, and we selected rice (*Oryza sativa* L.) as a model species because of its relevance to global food security. The data provided will not only allow the rice microbiome to be subjected to in-depth analyses, but also to be linked to other plant microbiomes in the future.

**Fig. 1:**
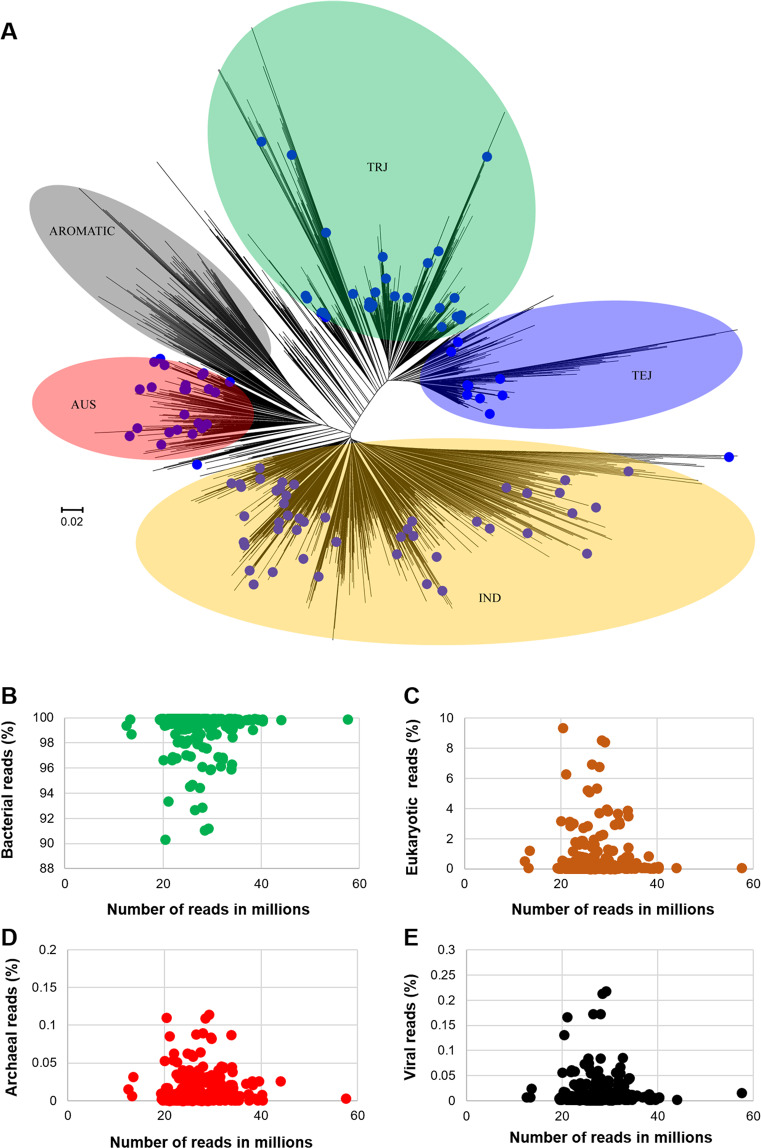
Phylogenetic tree of the implemented rice genotypes and overall composition of the metagenomic datasets. The phylogenetic tree was constructed with genetic data from the Rice Diversity Panel II core collection (C-RDP-II). Genotypes that were included in the present study are labelled with blue dots (**A**). TRJ: tropical japonica, TEJ: temperate japonica, IND: indica. The scatter plots show the number of microbial and viral reads in the obtained metagenomes (**B**: bacteria, **C**: eukaryota, **D**: archaea and **E**: viruses) according to Kraken2 classification.

**Fig. 2:**
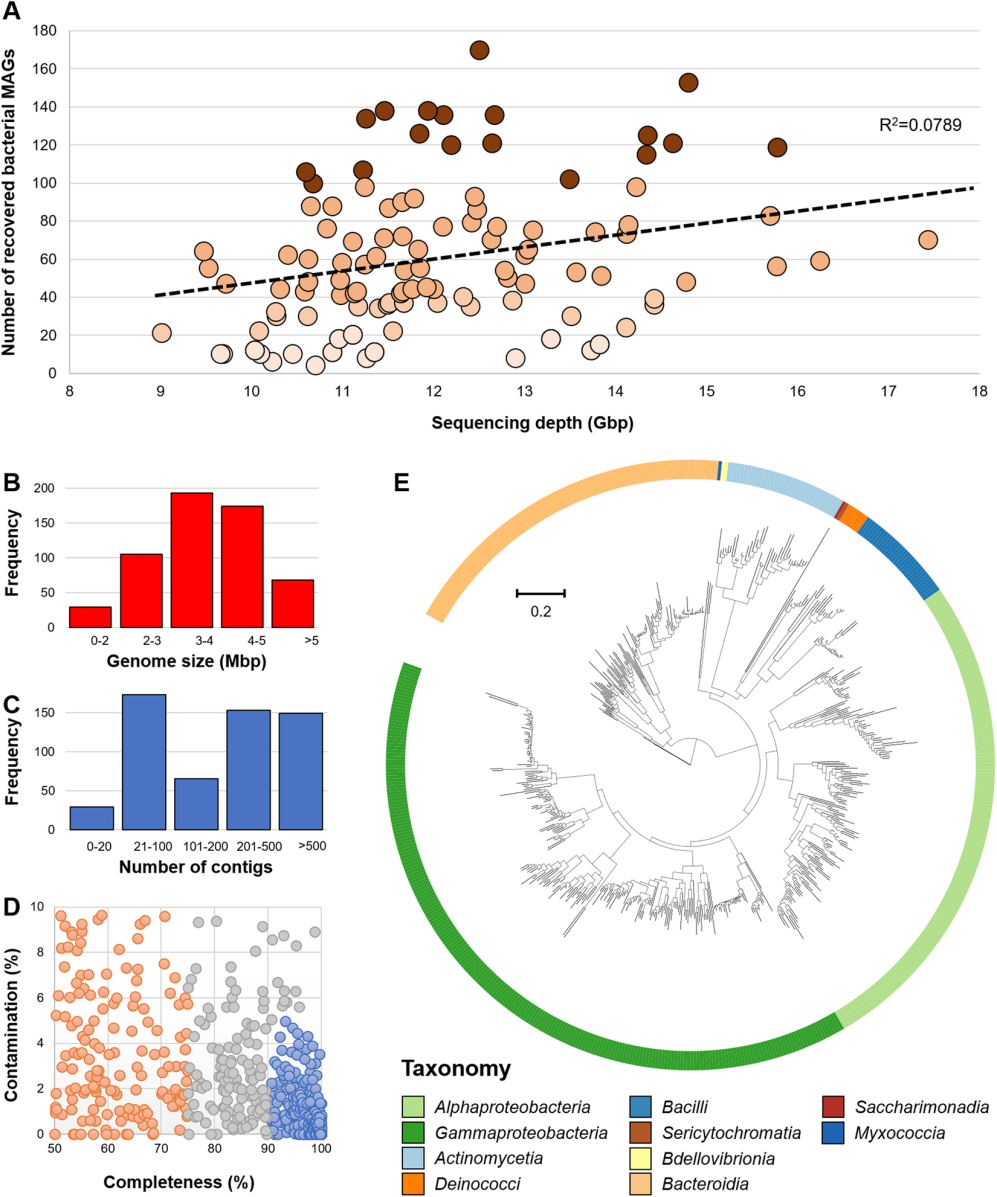
Recovery of metagenome-assembled genomes (MAGs) from 110 rice genotypes. MAGs were recovered from each rice genotype by using a combination of binning methods. The number of recovered MAGs per rice cultivar was plotted against the sequencing depth in order to determine the interrelation of the two variables (**A**). Quality metrics are shown for the recovered 569 MAGs with medium quality according to the minimum information metagenome-assembled genome (MIMAG) standards (**B**–**D**). A phylogenetic tree was constructed to visualize the diversity of the recovered MAGs (**E**). Different bacterial taxonomic groups (class level) are highlighted with different colours in the phylogenetic tree.

Here we present datasets derived from the phyllosphere metagenomes of 110 rice (*Oryza sativa*) genotypes (Fig. 1A). A total of 1.34 terabases (Tb) of metagenomic reads were generated with an average sequencing depth of 4.06 Gb/sample. After quality filtering, a total of 17.8 billion high-quality reads was retained. Therein, 99.04% of the reads were classified as reads originating from bacteria (min: 90.4%, max: 99.9%; Fig. 1B). The remaining reads were assigned to eukaryotes (average: 0.568%, min: 0.006%, max: 9.047%), viruses (average: 0.014%, min: 0.001%, max: 0.218%), and archaea (average: 0.013%, min: 0.0%, max: 0.114%) (Fig. 1C–E). By implementing three binning methods (Maxbin2, MetaBAT2, and Vamb) a total of 6,705 MAGs was recovered. On average, 60.9 MAGs were recovered per rice cultivar with a minimum of 4 and a maximum of 170 MAGs (Fig. 2A). A low interrelation between sequencing depth and the number of recovered MAGs was found (R^2^ = 0.074). A total of 569 non-redundant MAGs with a completeness higher than 50% and contaminations less than 10% was obtained (Fig. 2D, Supplementary Dataset 2). Within this set, 289 MAGs were classified as high-quality draft MAGs (completeness higher than 90% and contaminations lower than 5%). An overview of the assembly statistics for the 569 medium-quality draft MAGs including genome size, number of contigs, completeness, and contamination is provided in Fig. 2B–D. A major proportion of the MAGs was assigned to the bacterial classes *Alphaproteobacteria* (n = 153), *Gammaproteobacteria* (n = 226), and *Bacteroidia* (n = 106; Fig. 2E). All of the predominant bacterial classes represent common colonizers of the plant phyllosphere. The remaining MAGs were assigned to the bacterial classes *Bacilli* (n = 32), *Actinomycetia* (n = 37), *Deinococci* (n = 7), *Saccharimonadia* (n = 4), *Bdellovibrionia* (n = 2), *Sericytochromatia* (n = 1), and *Myxococcia* (n = 1). A total of 235 MAGs could not be classified at species level. Of these MAGs, five MAGs were only assignable at family level which indicates potential occurrence of yet unknown taxa.

Our approach has resulted in a representative dataset covering the phyllosphere microbiomes of 110 rice cultivars^10^. It is currently the largest approach to recover MAGs from a single plant species and thus can provide novel cues to better understand microorganisms that colonize this widespread and important micro-environment. The dataset was obtained from plants that were all grown in one geographical region where they were exposed to specific climatic conditions. Therefore, the influence of environmental factors such as rainfall and temperature fluctuations on microbial community composition remains to be further explored in targeted approaches. The phyllosphere microbiome is known to respond dynamically to climatic conditions, so it is expected that major changes in bacterial and fungal populations will occur as they change. Overall, the presented data resource may provide a valuable basis for future ecological studies, biotechnological mining approaches, and comparative assessments with genomes or MAGs from other plant microbiome studies.

## Methods

### Selection of rice genotypes

The implemented rice varieties were selected from the Rice Diversity Panel II core collection (C-RDP-II) that was established by the International Rice Research Institute (IRRI;^11^. This collection was employed in order to ensure that the study is performed on a representative set of *Oryza sativa* genotypes. The C-RDP-II contains 584 rice accessions that are genotyped with 700,000 single-nucleotide polymorphism (SNP) markers. From these, a subset of 110 cultivars was selected based on their phylogenetic distribution (Fig. 1A). The same cultivars were used in a forgoing study to phenotype them in terms of their resistance to rice blast disease^12^. The seeds used for planting the experimental field were propagated by the Rice Research Center, Guangdong Academy of Agricultural Sciences. A phylogenetic tree was constructed with MEGA4^13^ to visualize the position of the selected rice genotypes within the C-RDP-II collection; the underlying matrix is provided in Supplementary Dataset 3 to facilitate correlation analyses.

### Design of the field experiment

The experimental field site was located in the village Luoxi in Taojiang County, which is located in the northwest of Hunan Province, China (28°38′09″ N, 112°0′57″ E). Hunan Province has a temperate, humid subtropical monsoon climate that is ideal for rice cultivation. The fields at the site were continuously planted with rice plants of various cultivars for the last 20 years. Throughout this period, the field site was operated by the Hunan Identification Center for Rice Blast Disease Resistance. Rice plants were grown during the typical period for conventional production (one planting per season) in this geographic region. The rice field was pretreated with a basic chemical fertilizer 10 days before planting the seedlings (seedling transplantation date: July 5^th^, 2019). Commercial ammonium bicarbonate (N content: 17.1%; executive standard: GB/T3559–2001) produced by Hubei Xingshengyuan Bio-engineering Co., Ltd. was used as basic chemical fertilizer at 0.075 kg/m^2^. The field was plowed on the day of the planting. Urea fertilizer (N content: 46%; executive standard: GB/T2440–2017) produced by Shandong Luhengsheng chemical industry Co., Ltd. was used as top dressing at 0.015 kg/m^2^. The top dressing was applied three times during the experiment. Applications were conducted 7 d ahead of seedling transplantation, 14 d after seedling transplantation, and at the main tillering stage. The field experiment design followed a completely randomized block design (Supplementary Dataset 4). Three blocks were planted with respectively 110 genotypes in separate plots; each plot served as a replicate with a total of three plots for each rice genotype. The plots were transplanted with 100 seedlings at a plant density of 25 seedlings/m^2^. The plots in each block were separated by a ditch with a width of 50 cm and a depth of 10 cm. All plots shared the same nutrient and water management.

### Sampling and processing of plant material

Rice leaves were harvested at the booting stage (September 5^th^, 2020). The average temperature (10-day average) was 21.4 °C and the average relative humidity (10-day average) was 93.7% before the sampling; no rainfall was recorded during this time period. In each plot, leaf samples were collected from plants selected by implementing a five-point sampling method. Five plants were collected from each point. The second and third leaves from the top of each plant, representative for areas that can be affected by foliar diseases of rice, were removed with the stalk. The detached leaves were immediately wrapped with a sterilize gauze to minimize the leakage of leaf tissue fluid, which might contaminate the leaf samples with plant organelles and microbial endophytes. They were kept in cooling boxes at 4 °C. Leaf samples from each rice cultivar were subsequently pooled together as one combined sample for bacterial enrichment. The whole harvesting process was completed from 9 am to 11 am to ensure that the temperature and relative humidity were comparable. After leaf sampling, samples were transported to a nearby laboratory (Hunan Academy of Agriculture Science, Plant Protection Institute) for further processing. A total of 5 g leaf material from each rice cultivar was used to enrich bacteria from the plant phyllosphere. The leaf samples for each replicate were transferred into a 250-mL conical flask containing 100 mL sterile PBS buffer (0.02 M, pH 7.0) and 100 μL Tween-30. The flask was placed in a shaker for 1 h set at 200 rpm/min and then sonicated for 5 min at a frequency of 30 kHz, while a temperature of 4 °C was maintained. After sonication, the leaves were recycled and treated with the same procedure two more times to ensure that the bacterial cells were thoroughly washed off from the leaf surface. The suspensions from the washing steps were pooled together and subjected to centrifugation (1,500 rpm/min, 1 min, 4 °C). The supernatant was then collected and again subjected to centrifugation (12,000 rpm/min, 15 min, 4 °C). The pellets obtained after the second centrifugation were stored at −80 °C before further use.

### Extraction of metagenomic DNA and high-throughput sequencing

The frozen pellets were thawed on ice prior to DNA extraction. Total community DNA extraction was conducted using the MagPure DNA LQ Kit (Reagent serial number: Cat. No. D6356–02, Reagent batch number: Lot No. 20200304; OE Biotech Co. Ltd., Shanghai, China), following the manufacturer’s instruction. Further processing for metagenomic sequencing was performed by OE Biotech Co. Ltd., Shanghai, China. DNA library preparation was conducted using a TruSeq Nano DNA LT Sample Preparation Kit (Illumina; United States). The obtained libraries were then sequenced in three technical replicates using the NovaSeq. 6000 system (Illumina; United States) and 2 × 150 bp paired-end sequencing.

### Taxonomic assignments based on short metagenome reads

**Fig. 3:**
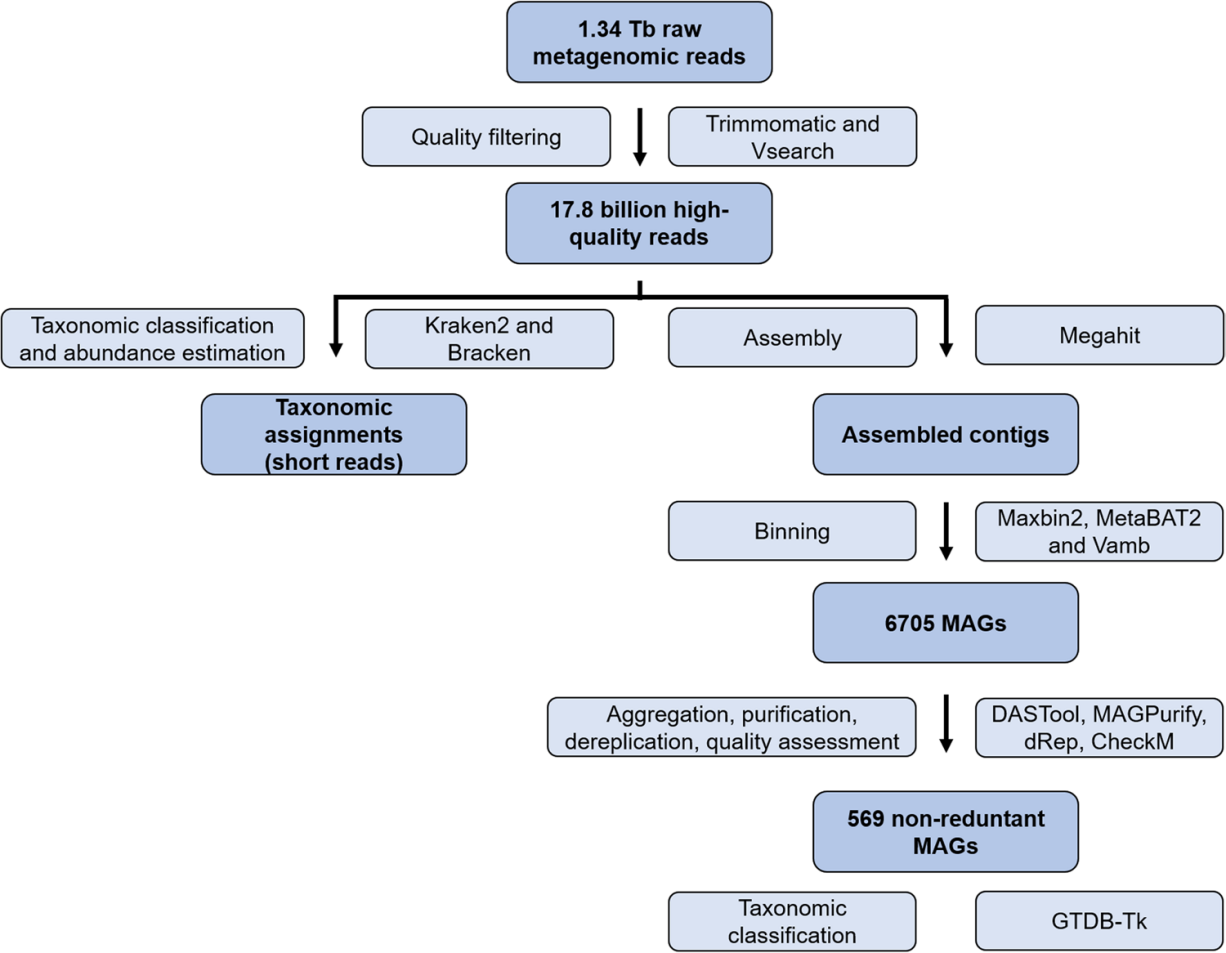
Overview of the bioinformatic workflow for the recovery of metagenome-assembled genomes (MAGs) from the phyllosphere metagenomes of 110 rice (*Oryza sativa* L.) genotypes. Process steps and the corresponding bioinformatic tools are indicated in light blue while the resulting data is indicated in dark blue.

The overall bioinformatic workflow conducted for this study is visualized in Fig. 3. Quality filtering was performed using Trimmomatic v0.39^14^ and VSEARCH v2.14.2^15^ to remove Illumina sequencing adaptors and to perform initial quality filtering (removal of low-quality reads; Phred < 20). Quality-filtered reads were used as inputs for microbial community analysis using Kraken2 v2.0.9^16^. Kraken2 classifies individual metagenomic reads by mapping all *k*-mers to the lowest common ancestor (LCA) of all reference genomes^16^. The standard Kraken2 database was used for classification of all datasets; it contains bacterial, archaeal, and viral domains, along with the human genome and a collection of common vectors. Following Kraken2 analysis, species abundances were estimated using Bracken v2.6.0^17^.

### Metagenome assembly and reconstruction of bacterial metagenome-assembled genomes

Different binning methods including Maxbin2 v2.2.7, MetaBAT2 v2.12.1, and Vamb v3.0.2^18–20^ were used to reconstruct metagenome-assembled genomes (MAGs). The binning approaches based on Maxbin2 and MetaBAT2 were conducted using individual sample replicates whereas the binning using Vamb was performed using multisplit approaches by concatenating individual assembled contigs from each replicate within a sample. Multiple bins recovered with these binning methods were aggregated using DASTool v1.1.1^21^ with the parameter:–score_threshold 0.3. Bins were further refined using MAGPurify v1.0^22^ to remove contaminations from genome bins. Metagenome-assembled genomes were then dereplicated using dRep v2.2.3^23^ to obtain a non-redundant MAG set. Finally, the quality of MAGs was assessed using CheckM v1.0.13^24^.

### Taxonomic classification of bacterial metagenome-assembled genomes

Only medium-quality MAGs according to the current definition of the minimum information metagenome-assembled genome (MIMAG) standards^25^ were kept for further analyses. Taxonomical information of each MAG was obtained using GTDB-Tk v1.4.1^26^. A phylogenetic tree was constructed using PhyloPhlAn v3.0^27^. Subsequently, the phylogenetic tree was visualized using the interactive tree of life software (iTOL^28^).

## Data Records

The whole rice phyllosphere metagenome project was deposited in the European Nucleotide Archive (ENA) database under the study number PRJEB45634^29^. Shotgun metagenome reads and metagenome-assembled genomes were deposited under accession numbers ERS6595503-ERS6595833 and ERS6626560-ERS6627345, respectively. Details related to the rice cultivars and accession numbers for the retrieval of their metagenomes from public repositories are provided in Supplementary Dataset 1. Details related to all recovered MAGs and the accession numbers for their retrieval from public repositories are provided in Supplementary Dataset 2.

## Technical Validation

Potential cross-contamination of samples was limited using aseptic techniques. Enrichment of bacterial cells via sonication was repeated two times to ensure that the present were thoroughly washed off from the leaf surface. The metagenome-assembled genome (MAG) catalogue includes only those genomes that met specific quality thresholds, i.e. specified completeness and contamination levels according to CheckM v1.0.13^24^.

## Supplementary information

Supplementary Dataset 1

Supplementary Dataset 2

Supplementary Dataset 3

Supplementary Dataset 4

## Data Availability

No custom scripts were used to generate or process this dataset. Software versions and non-default parameters used have been appropriately specified where required.
